# Immunity to Human Immunodeficiency Virus (HIV) Infection

**DOI:** 10.1155/2012/629356

**Published:** 2012-07-05

**Authors:** Carlo Torti, Mirko Paiardini, Andrea Gori

**Affiliations:** ^1^Institute of Infectious and Tropical Diseases, University of Brescia, 25123 Brescia, Italy; ^2^Unit of Infectious Diseases, “Magna Graecia” University, 88100 Catanzaro, Italy; ^3^Department of Pathology, School of Medicine, Emory University, Atlanta, GA 30322, USA; ^4^Division of Infectious Diseases, San Gerardo Hospital, University of Milano-Bicocca, 20900 Monza, Italy

The advent of highly active antiretroviral therapy (HAART) in 1996 revolutionized life expectancy and quality of life of people infected by HIV in the developed Countries. Nowadays, HIV-infected patients can positively think that their life expectancy is comparable to that of people of the same age and with the same pattern of risk factors Indeed, there are clear demonstrations that mortality patterns in most nonintravenous drug users HIV infected individuals with high CD4+ T-cell counts restored by HAART are similar to those in the general population [1]. Unfortunately, however, not all HIV-infected patients on HAART are able to restore their CD4+ T-cell count, and signs of immune deficits or immune activation persist in most individuals despite an apparent control of HIV viremia and a mere increase of the CD4+ T-cell number. This persistent immune deregulation has been correlated, and it is believed to be—at least in part—in the causal pathway of end-organ diseases (such as non-AIDS-defining neoplasias or cerebrovascular events) that continue to affect our patient population. So, biological age of our patients has been estimated to be 10–15 higher than the current age for the risk of these complications [2]. It is therefore important to better understand risk factors and immune correlates of such persistent immune de-regulation, how to measure and how to correct it. For this reason, immune modulator strategies are eagerly awaited as a complement to HAART. In this special issue on immunity to HIV, we provide high quality papers that address these issues.

 The paper by S. C. Gaardbo et al. provides an in-depth review on possible reasons and mechanisms of an incomplete immune recovery. M. R. Pinzone et al. focuses on persistent immune replication in cellular or anatomical sanctuaries, how to measure such replication, and the need of new therapeutic strategies to target the latent viral reservoirs. To complete this scenario, Cotugno et al. contextualize these issues to vertically HIV-infected children. In addition, they describe immune responses to vaccinations as a means to detect and explain suboptimal reconstitution in these patients. Importantly, S. Duggal et al. underline the complex interactions between HIV, immune function, and nutrition. This paper merits consideration in light of the fact that the most HIV-infected patients reside in resource limited settings; malnutrition is a dangerous ally to HIV in these areas of the word, so we need a political commitment to fight this proudly.

 C. Tincati et al. studied a facet of persistent immune activation as a possible cause of suboptimal immune response. The authors observed that in vitro stimulation by lipopolysaccharide (LPS) produced a CD8+CD38+ upregulation in patients with smaller CD4+ T-cell recovery after HAART. This finding allowed the authors to speculate that the mere induction of CD38 on CD4+ cells may not entirely account for the immune activation induced by LPS in HIV-infected patients. Therefore, further studies are needed to better understand the regulation of T-cell activation. Rather simplistic theories may not be true.

HIV-associated neurocognitive disorder is an emerging complication in the era of HAART. It can be explained, at least in part, by inadequate penetration of antiretroviral drugs across the blood brain barrier, but also immune activation or persistent viremia in the macrophage lineage cells in the brain may have a role. The paper by Airoldi et al. adds a piece to the puzzle of the relationship between immune activation parameters and HIV load in the cerebrospinal fluid (CSF) of patients with HIV-associated neurocognitive disorders (HANDs). The main finding is that immune activation is hyperexpressed in patients with HAND and less sensitive to HAART than the HIV load in the CSF. It has to be seen whether immune activation is a better means to monitor the effect of HAART in these patients than the mere quantification of HIV RNA. So we probably need a multidimensional algorithm for diagnosis and followup of HAND including virological methods, immunological tests, neuroimaging techniques, and neuropsychological tests. Moreover, it is interesting to note that what happens in the peripheral blood does not fully account for what it is happening in the central nervous system as a sanctuary. This may be important and relevant for diagnosing and monitoring complications in this compartment.

Further two papers deal with B-cell responses, a topic that has been investigated not so in depth as T-cell responses. The review by Poudrier et al. concludes that host capacity to maintain dendritic cell homeostasis at mucosal sites, and therefore an effective B-cell response against HIV, have a key role to better control the infection. In their original work, M. Fogli at al. noted a dramatic expansion of exhausted tissue-like memory B cells related to an increase of Torque teno virus (TTV) which was assumed to reflect a functional competency of the immune function. Interestingly, such a defect was noted even in patients with a quite preserved CD4+ T-cell count (>350/mm^3^), possibly reinforcing the need of an earlier treatment.

Lastly, it is clear that HAART is not sufficient to entirely restore the immune function damaged by HIV. So, vaccine and immune-modulatory therapy should be experimented. The paper by Li et al. used a DNA prime/fowlpox virus boost regimen to immunize rhesus macaques. This study showed that the vaccine was able to induce a strong immune response and increase the CD4/CD8 ratio in the vaccinated animals. Ries et al. provided an extensive review on blocking type I interferon production as a means to reduce immune deregulation. This concept should clearly be taken into consideration as an opportunity for adjuvant immune-modulatory therapies in HIV infection.

In conclusion, this special issue encompasses a broad range of cutting-edge topics in HIV research. We feel that they will enrich the current body of the literature significantly.

*Carlo Torti* *Mirko Paiardini* *Andrea Gori*
